# Potentiation of Growth Inhibitory Responses of the mTOR Inhibitor Everolimus by Dual mTORC1/2 Inhibitors in Cultured Breast Cancer Cell Lines

**DOI:** 10.1371/journal.pone.0131400

**Published:** 2015-07-06

**Authors:** Euphemia Y. Leung, Marjan Askarian-Amiri, Graeme J. Finlay, Gordon W. Rewcastle, Bruce C. Baguley

**Affiliations:** 1 Auckland Cancer Society Research Centre, University of Auckland, Grafton, Auckland, New Zealand; 2 Department of Molecular Medicine and Pathology, University of Auckland, Grafton, Auckland, New Zealand; Taipei Medical University, TAIWAN

## Abstract

The mammalian target of rapamycin (mTOR), a vital component of signaling pathways involving PI3K/AKT, is an attractive therapeutic target in breast cancer. Everolimus, an allosteric mTOR inhibitor that inhibits the mTOR functional complex mTORC1, is approved for treatment of estrogen receptor positive (ER+) breast cancer. Other mTOR inhibitors show interesting differences in target specificities: BEZ235 and GSK2126458 are ATP competitive mTOR inhibitors targeting both PI3K and mTORC1/2; AZD8055, AZD2014 and KU-0063794 are ATP competitive mTOR inhibitors targeting both mTORC1 and mTORC2; and GDC-0941 is a pan-PI3K inhibitor. We have addressed the question of whether mTOR inhibitors may be more effective in combination than singly in inhibiting the proliferation of breast cancer cells. We selected a panel of 30 human breast cancer cell lines that included ER and PR positive, HER2 over-expressing, and “triple negative” variants, and determined whether signaling pathway utilization was related to drug-induced inhibition of proliferation. A significant correlation (*p* = 0.005) was found between everolimus IC_50_ values and p70S6K phosphorylation, but not with AKT or ERK phosphorylation, consistent with the mTOR pathway being a principal target. We then carried out combination studies with four everolimus resistant triple-negative breast cancer cell lines, and found an unexpectedly high degree of synergy between everolimus and the other inhibitors tested. The level of potentiation of everolimus inhibitory activity (measured by IC_50_ values) was found to be cell line-specific for all the kinase inhibitors tested. The results suggest that judicious combination of mTOR inhibitors with different modes of action could have beneficial effects in the treatment of breast cancer.

## Introduction

mTOR (mammalian target of rapamycin) is a component of two distinct cell signaling complexes, mTOR complex 1 (mTORC1) and mTOR complex 2 (mTORC2), each of which plays an essential role in the control of cell proliferation. Activating mutations in *PIK3CA* deregulate the PI3K/AKT/mTOR pathway and are frequent in breast cancer [1]. Furthermore, patients whose tumors have *PIK3CA* mutations show higher response rates to PI3K/AKT/mTOR inhibitors than do patients whose tumours lack *PIK3CA* mutations [2], suggesting that this signaling pathway is a promising therapeutic target for this disease [3].

A number of inhibitors of the PI3K/AKT/mTOR pathway have now been identified. Everolimus, an allosteric mTORC1-specific inhibitor, has been used clinically to treat ER+ breast cancer [4,5]. KU-0063794 [6] and AZD8055 [7,8] are ATP competitive mTOR inhibitors that target both mTORC1 and mTORC2 complexes. AZD8055 inhibits the phosphorylation of mTORC1 substrates p70S6K and 4E-BP1 as well as phosphorylation of the mTORC2 substrate AKT and downstream proteins [8]. AZD8055 has not been advanced to clinical use, due to an unfavorable toxicity profile relative to those of other rapalogs, which includes the elevation of serum transaminase concentrations [9]. A follow-up compound, AZD2014 [10] is currently being investigated in phase I trials (NCT01597388) and no rise in transaminases has been reported [9]. Through compound optimization, the newly discovered AZD3147 [11] is an extremely potent and selective dual inhibitor of mTORC1 and mTORC2 with potential for further development as a clinical candidate. BEZ235 and GSK2126458 [12–14] are dual PI3K/mTOR catalytic inhibitors that can potently inhibit AKT S473 phosphorylation [15,16]. Similar AKT S473 inhibiting activity is observed with a pan-PI3K inhibitor GDC-0941 [17]. The mTOR blockade with everolimus may result in the activation of compensatory feedback loops with an increase in the activated phosphorylated form of AKT (pAKT), that would in turn result in decreased efficacy [18].

**Fig 1:**
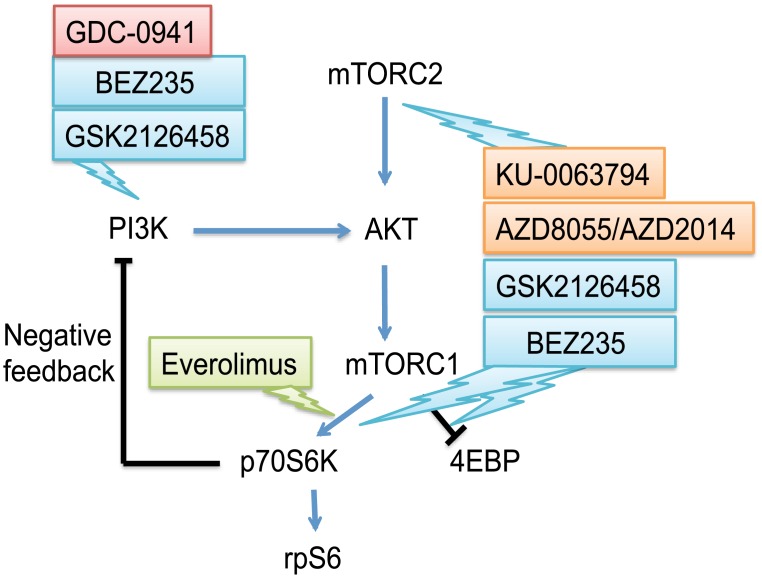
Schematic representation of a network of PI3K/AKT/mTOR signaling. Inhibitors targeting the pathways are used in this study. Blue arrows and black lines represent activating and inhibitory connections, respectively. GDC-0941, pan-PI3K inhibitor; BEZ235 and GSK2126458, dual PI3K/mTOR kinase inhibitors; AZD8055, AZD82014 and KU-0063794, dual mTORC1/mTORC2 kinase inhibitors; and everolimus, allosteric mTORC1 inhibitor.

We have chosen everolimus to address the question of whether anti-proliferative activity is related to inhibition of the mTOR signaling pathway, as measured by p70S6K phosphorylation. We have utilized 30 human breast cancer cell lines, including those that could be classified as ER and PR positive, HER2 over-expressing, or “triple negative”. We have also investigated whether the dual PI3K/mTOR inhibitors BEZ235 and GSK2126458, or the dual mTORC1/mTORC2 inhibitor (AZD8055), can sensitize everolimus resistant breast cancer cell lines to everolimus by reducing AKT phosphorylation. We selected four everolimus resistant triple-negative breast cancer cell lines (MDA-MB-231, MDA-MB-436, BT20 and HCC1143) to answer the question of whether ATP competitive mTORC1/2 inhibitors synergize with everolimus in their effects on cell proliferation. We have tested for possible interactions between everolimus and both dual PI3K/mTOR inhibitors and the pan-PI3K inhibitor GDC-0941 (Fig 1). These experiments help to answer the important question of whether combination therapy can overcome everolimus resistance.

## Material and Methods

### Cell culture

Culture conditions have been described in detail previously [19,20]; MCF-7, T47D, SKBr3, MDA-MB-468, BT20, MDA-MB-231, HCC1143, HCC70, SUM149PT, HCC1954, MDA-MB-436, HPL100, BT549 and SUM159PT cells were purchased from the American Type Culture Collection (ATCC). PMC42ET [21] was a kind gift from Dr. Chanel Smart (The University of Queensland). SUM149PT and SUM150PT were grown in Ham's F-12 with 5% fetal bovine serum (FBS) supplemented with 5 μg/mL insulin, 1 μg/mL hydrocortisone, 10 mM HEPES (pH 7.4) and penicillin/streptomycin (100 U/ml and 100 μg/ml, respectively). Apart from MCF-7 sub-lines, all other cells were grown in α-MEM containing 5% FBS. All the other growth media contained insulin/transferrin/selenium supplement, added according to the manufacturer’s instructions (Roche), as well as penicillin/streptomycin (100 U/ml and 100 μg/ml, respectively). The TamR7 cell line was established by culturing MCF-7 cells in the presence of progressively increasing concentrations of tamoxifen (0.1–3 μM; stock dissolved in DMSO) and then maintaining them for >15 months in 3 μM tamoxifen. The TamR3 and TamR6 cell lines were generated by growth of MCF-7 cells in phenol-red-free RPMI containing 10% charcoal-stripped fetal bovine serum (Invitrogen, Auckland, NZ), over a period of 3 months in progressively increasing concentrations of tamoxifen (1 nM to 1 μM in ethanol) and then maintaining them for >15 months in 1 μM tamoxifen. The TamC3 and TamC6 cell lines were generated by exposure of MCF-7 cells for >16 months to the above growth medium but lacking tamoxifen [16,19,22]. The FulvR1a, FulvR1c and FulvR2a cell lines were generated by growth of MCF-7 cells in phenol red-free RPMI containing 5% charcoal-stripped fetal bovine serum, over a period of 3 months in progressively increasing concentrations of fulvestrant (1 nM to 100 nM; stock dissolved in ethanol), and then maintaining them for >12 months in 100 nM fulvestrant. The FulvC1a, FulvC1b and FulvC2 cell lines were generated by exposure of MCF-7 cells for >12 months to the above growth medium but lacking fulvestrant [23]. All experiments were carried out in cells grown in their respective growth media but without tamoxifen or fulvestrant.

### Chemicals and reagents

Tamoxifen was purchased from Sigma (Auckland, NZ). Everolimus, AZD8055 and AZD2014, KU-0063794 and GDC-0941 were purchased from Selleck Chemicals (Houston, USA). BEZ235 [24,25] and GSK2126458 [12] were synthesized according to published protocols.

#### Cell proliferation assay

As described in detail previously [20], proliferation was measured using a thymidine incorporation assay. Cells were seeded at 3000 per well in 96 well plates in the presence of varying concentrations of inhibitors for 3 days. ^3^H-thymidine was added to each well and incubated for 6 h; the cells were harvested on glass fiber filters using an automated TomTec harvester. Filters were incubated with Betaplate Scint and thymidine incorporation measured in a Trilux/Betaplate counter. Effects of inhibitors were determined relative to the incorporation of ^3^H-thymidine into DNA of control (non-drug-treated) cells.

#### Cell viability assay

Cells were seeded as described in cell proliferation assay in the presence of varying concentration of inhibitors for 3 days. AlamarBlue (Life Technologies) cell viability reagent was used to assess cell viability by adding 10% of the sample volume into breast cancer cells in culture media, followed by a 3 hours incubation at 37°C. The resulting fluorescence was assessed on a fluorescence plate reader with excitation at 570 nm and fluorescence emission was read at 585 nm.

#### Western blotting

As described in detail previously [20], breast cancer cell lines were grown to log-phase, washed twice with ice-cold PBS, and lysed in SDS lysis buffer according to the manufacturer’s protocol (Cell Signaling Technology, Danvers, MA). Protein concentration was quantified using the bicinchoninic acid reagent (Sigma). Cell lysates containing 25 μg of protein were separated by SDS-polyacrylamide gel electrophoresis (PAGE), and transferred to PVDF membranes (Millipore). Membranes were immunoblotted with antibodies against phospho-AKT (S473), total AKT, phospho-p70S6K (T389), total p70S6K, phospho-rpS6 (S235/236), total rpS6, phospho-4E-BP1 (T70), total 4E-BP1, phospho-ERK (T202/Y204), total ERK (all from Cell Signaling Technology), tubulin (Sigma), and actin (Millipore). Bound antibody was visualized using SuperSignal West Pico (Thermo Scientific, Waltham, MA) or ECL plus (GE Healthcare, Auckland, NZ) and the chemiluminescence detection system by Fujifilm Las-3000. Densitometry was performed using ImageJ. The relative intensities of phosphorylated proteins were normalized using either tubulin or actin as the standard. The fold change was then calculated between MCF7 and BT20 as standard reference.

#### Flow cytometry

As described in detail previously [16], cells (1 × 10^6^ cells) were grown in 3.5 cm Petri dishes and incubated with inhibitors for 24 h. They were harvested, washed with 1% FCS/PBS, resuspended in 200 μl of PBS, fixed in 2 ml of ice-cold 100% ethanol and stored overnight at −20°C. The cells were washed and resuspended in 1 ml of 3% FCS/PBS containing RNase (1 μg/ml) and propidium iodide (PI) (10 μg/ml) for 30 min at room temperature. DNA content was determined using forward scatter (FSC) intensity by PI staining based on a total 30,000 acquired events by FACScan cytometry.

### Data analysis

The Bliss additivism model [26] was used to classify the effect of combining everolimus and other inhibitors as additive, synergistic, or antagonistic. A theoretical curve was calculated for combined inhibition using the equation *E*bliss = *E*A + *E*B - *E*A x *E*B, where *E*A and *E*B are the fractional inhibitions obtained by drug A alone and drug B alone at specific concentrations. *E*bliss is the fractional inhibition that would be expected if the combination of the two drugs was exactly additive. The differences (Bliss) in the experimentally measured fractional inhibition (*E*xpt) and *E*bliss are defined as synergism (*E*xpt < *E*bliss), additivity (*E*xpt = *E*bliss), and antagonism (*E*xpt > *E*bliss). Bliss = 0 would indicate the combination is additive; Bliss > 0 would indicate the percentage increase in maximal inhibition above additivity (synergy); and Bliss < 0 would indicate the percentage decrease in maximal inhibition below additivity (antagonism).

As described in detail previously [20], data were analyzed using a one-way ANOVA coupled with multiple comparisons versus treatment control applying the Holm-Sidak method correction, where p < 0.05 denotes a statistically significant difference. T-test or Mann-Whitney Rank Sum Test was used for comparison between two groups. Correlation analysis was performed with Linear Regression analysis or Spearman’s rank correlation coefficient (*R)* and statistical significance (*P)* using SigmaPlot. Values of *P*<0.05 were considered to be statistically significant.

## Results

### Responses to everolimus in a panel of breast cancer cell lines

**Fig 2:**
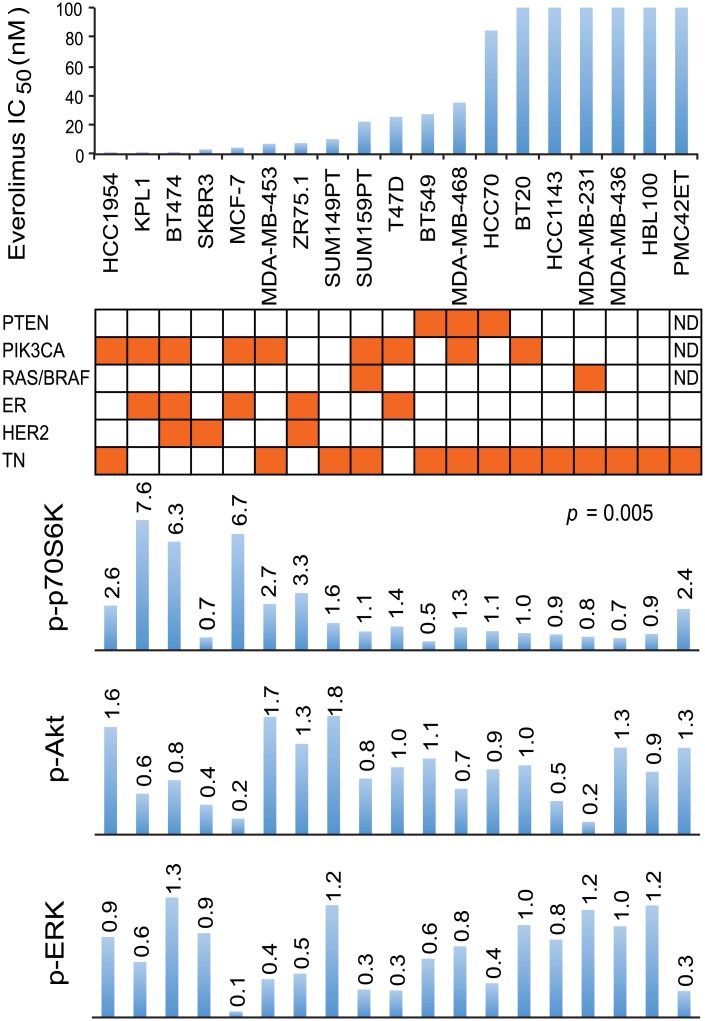
Relationship between drug sensitivity, mutation status, receptor status and pathway utilization. IC_50_ values for everolimus are represented on the y-axis and individual cell lines on the x-axis. Orange shading in the matrix below indicates PTEN, PIK3CA, RAS and BRAF mutations (Roche Cancer Genome Database 2.0 [45]), and identifies cell lines that are ER positive, HER2 positive and triple-negative (TN). Relative levels of phosphorylation of p70S6K, AKT and ERK from the respective breast cancer cell lines (untreated) are shown as bar graphs. Bands are normalized to tubulin control and bars represent changes in fold compared with BT20 and expressed as the mean of two experiments.

A series of breast cancer cell lines was assayed for sensitivity to everolimus (as measured by IC_50_ values) using a 3-day ^3^H-thymidine incorporation assay (Fig 2). A significant negative correlation (*p* = 0.005) was found between everolimus IC_50_ values and p70S6K phosphorylation, but not between everolimus IC_50_ values and AKT or ERK phosphorylation. Estrogen receptor positive breast cancer cell lines showed significantly higher sensitivity to everolimus than did receptor negative lines (*p* = 0.034). However, no significant correlation was observed between *PIK3CA* mutation status and either sensitivity to everolimus (*p* > 0.05) or degree of p70S6K phosphorylation (*p* > 0.05).

### Responses to everolimus in a panel of tamoxifen-resistant MCF-7 lines

**Fig 3:**
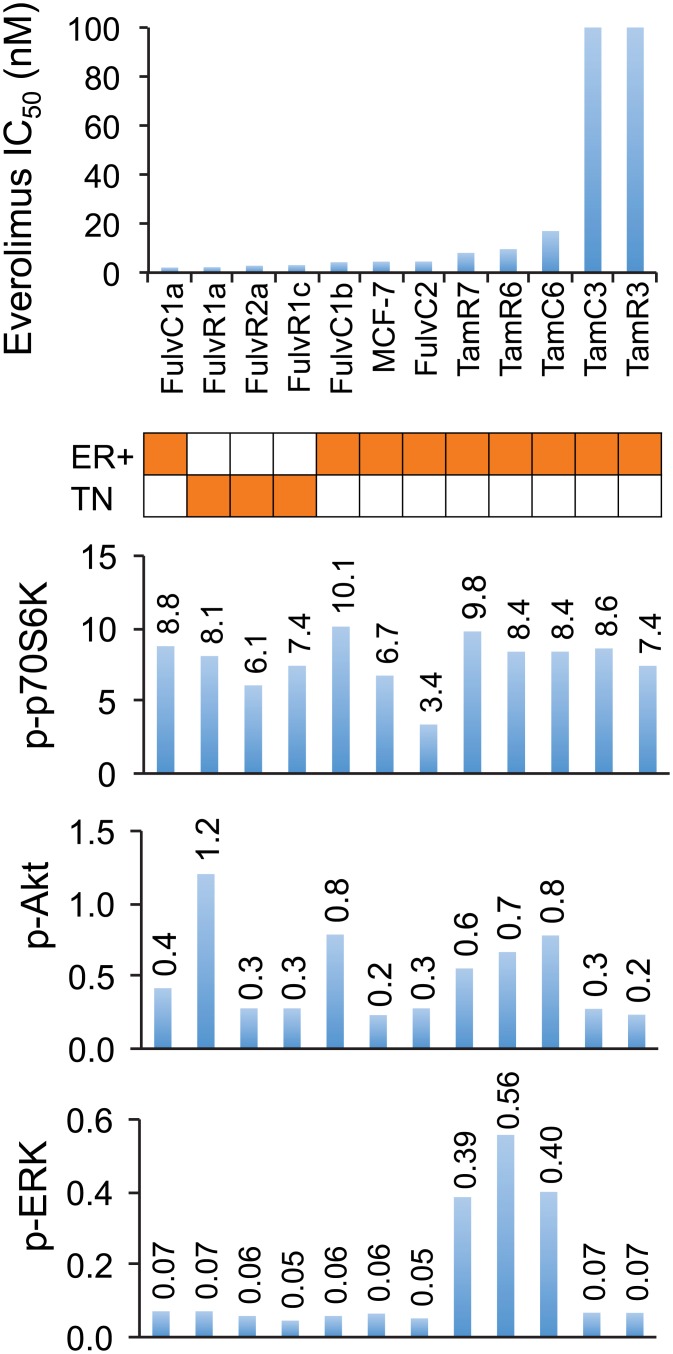
Relationship between drug sensitivity and pathway utilization in MCF-7 parental and sub-lines. IC_50_ values for everolimus are represented on the y-axis and individual cell lines on the x-axis. Orange shading in the matrix indicates estrogen receptor positive (ER), and triple-negative (TN) cell lines. Relative levels of phosphorylation of p70S6K, AKT and ERK of breast cancer cell lines are shown as bar graphs. Bands are normalized to tubulin or actin control and bars represent changes in fold compared with MCF-7 and expressed as the mean of two experiments.

The lack of correlation among cell lines with widely varying properties raised the question of whether there was a relationship between the level of p70S6K phosphorylation and proliferation inhibition by everolimus in a series of lines that had a common genetic background. Hence, we utilized a series of well characterized receptor positive (ER+) MCF-7 tamoxifen-resistant sub-lines [16,19,20,23,27], as well as a series of triple negative MCF-7 fulvestrant-resistant sub-lines [23] (Fig 3). The MCF-7 parental line and nine of its sub-lines were sensitive to everolimus with IC_50_ values of less than 20 nM, while two sub-lines TamC3 and TamR3 (both with IC_50_ > 100 nM) showed relative resistance. However, no significant correlation was observed between the degree of p70S6K phosphorylation, AKT phosphorylation, ERK phosphorylation and everolimus sensitivity (Fig 3).

### Tests for synergy using combinations of everolimus with BEZ235, GSK2126458 or AZD8055

**Fig 4:**
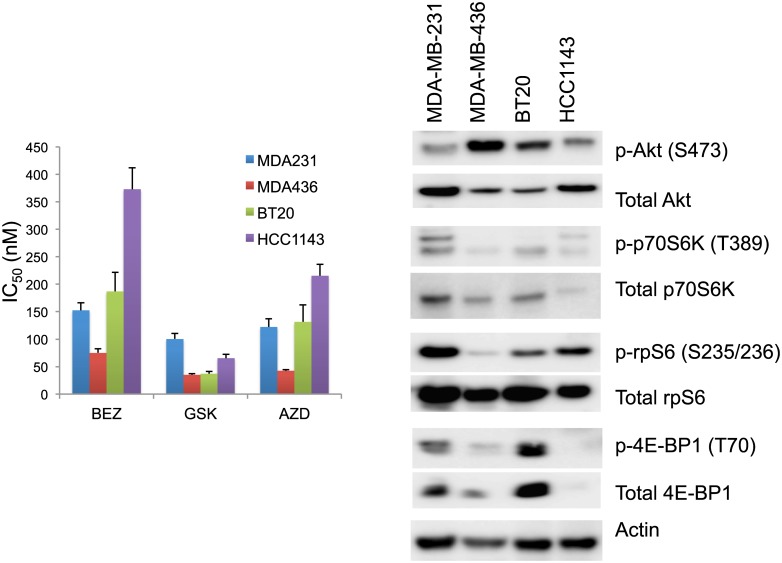
Signaling pathway usage and growth inhibitory response of MDA-MB-231, MDA-MB-436, BT20 and HCC1143 exposed to different drugs. (A) IC50 values (50% inhibition of thymidine incorporation) are shown for BEZ235 (BEZ), GSK2126458 (GSK) and AZD8055 (AZD). Cells were treated with drugs for 3 days and cell proliferation was measured by the thymidine incorporation assay. Results are shown as the mean ± standard error from triplicate experiments. (B) Signaling pathway usage as measured by basal protein phosphorylation of AKT, p70S6K, rpS6 and 4E-BP1 in the four cell lines. Immunoblots with antibodies specific for phosphorylated proteins and their respective total protein are indicated. Actin is the loading control.

We chose four everolimus resistant triple-negative breast cancer cell lines, MDA-MB-231, MDA-MB-436, BT20 and HCC1143, characterized by a diversity of activated signaling pathways and proliferation inhibition by ATP competitive inhibitors to mTOR. The cell lines were assayed for sensitivity to BEZ235, GSK2126458 and AZD8055, using a 3-day ^3^H-thymidine incorporation assay (Fig 4A). PI3K/AKT/mTOR pathway usage was analyzed by western blots (Fig 4B). MDA-MB-436 showed the highest AKT phosphorylation, relatively low phosphorylated rpS6, and had the lowest IC_50_ of the three inhibitors tested as compared to MDA-MB-231, BT20 and HCC1143.

**Fig 5:**
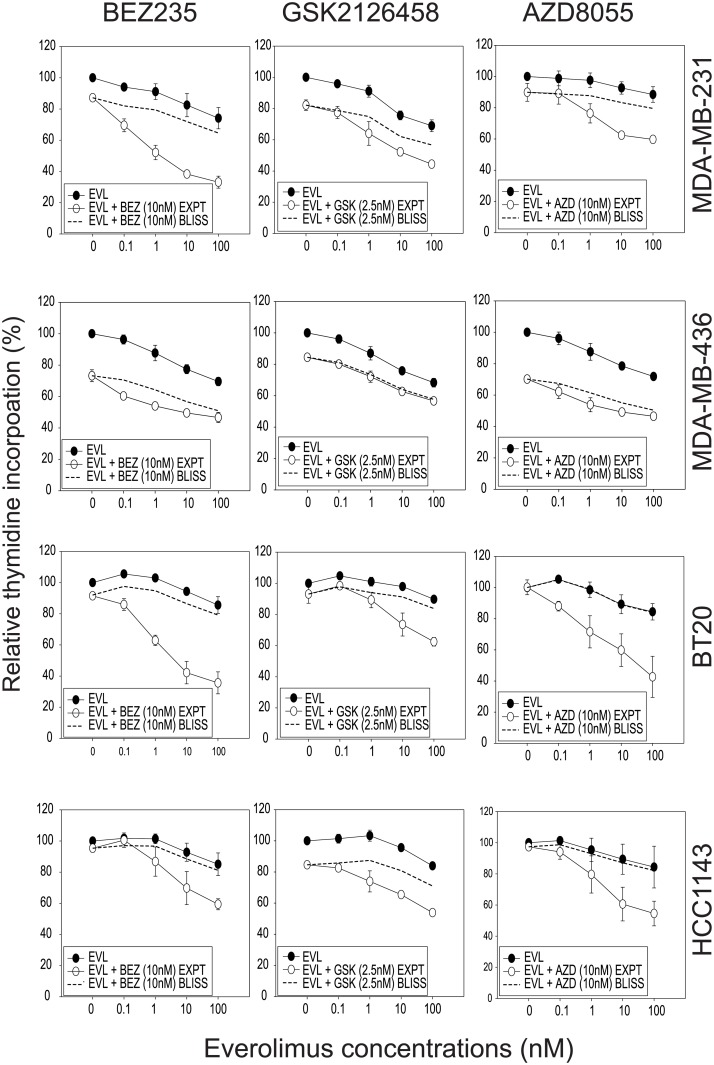
The growth inhibitory effects of drug combinations on MDA-MB-231, MDA-MB-436, BT20 and HCC1143 breast cancer cell lines. Growth inhibitory effects of combinations of everolimus with BEZ235 (BEZ) (left hand panel), GSK2126458 (GSK) (middle panel) and AZD8055 (AZD) (right hand panel) using the Bliss additivism method. Dashed line, Bliss additivity curve, representing the theoretical expectation if the combined effects of everolimus with kinase inhibitors were exactly additive. Averages of three independent experiments are shown.

We measured the effects of combinations of everolimus and mTOR ATP competitive inhibitors (BEZ235, GSK2126458, or AZD8055) (Fig 5). The Bliss additivism model [26] was used to assess drug interactions; this method has an advantage over combination index analysis since it is possible to evaluate the nature of drug interactions even when the maximal inhibition by mTOR inhibitors as single agents is too low for a reliable IC_50_ value to be obtained. The model indicates synergy between everolimus and mTOR ATP competitive inhibitors for all lines tested (Fig 5).

**Fig 6:**
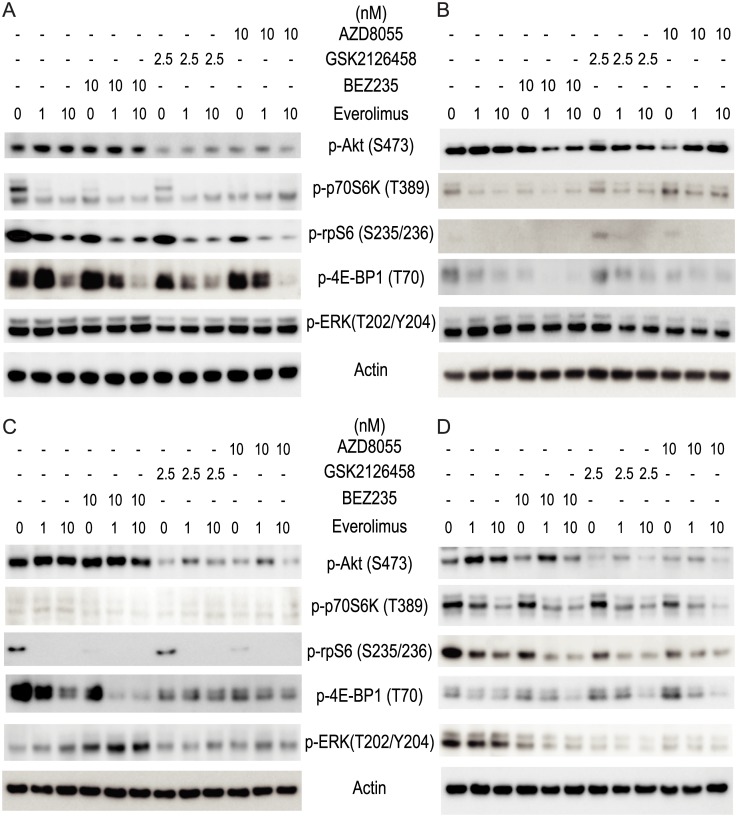
The cellular response to drug combinations of breast cancer cell lines. (A) MDA-MB-231, (B) MDA-MB-436, (C) BT20 and (D) HCC1143 breast cancer cells were treated with drugs for 24 h and signaling pathway usage was measured by protein phosphorylation of AKT, p70S6K, rpS6, 4E-BP1 and ERK in the four cell lines. Immunoblots with antibodies specific for phosphorylated proteins are indicated. Actin is the loading control.

The cellular responses to inhibitor combinations were also assessed by measuring phosphorylation of AKT, p70S6K, rpS6, 4E-BP1 and ERK (Fig 6; S1, S2 and S3 Figs). The degree to which thymidine incorporation was suppressed in the cell lines tested showed no correlation with the signaling responses (as measured by protein phosphorylation) induced by inhibitors either alone or in combination. Both GSK2126458 and AZD8055 reduced AKT phosphorylation in MDA-MB-231 and HCC1143 cells. BEZ235 showed no effect on the AKT phosphorylation, but showed the highest Bliss value (Bliss = 26±5, where Bliss > 0 indicates synergy) in proliferation assays (Table 1). Similar results were observed with the other cell lines tested. Increased ERK phosphorylation in BT20 treated with BEZ235 showed no correlation with drug sensitivity measured by H^3^-thymidine incorporation (Figs 5 and 6). Changes in signaling responses therefore did not reflect the synergistic effects on proliferation, regardless of whether the inhibitor targeted mTOR alone or both PI3K/mTOR.

**Table 1:** Bliss values for combinations of everolimus and PI3K/mTOR inhibitors in four cell lines. Synergy, as noted by a positive Bliss value, was observed in all the cell lines in the combinations of everolimus and PI3K/mTOR or mTOR inhibitors.

		MDA-MB-231	MDA-MB-436	BT20	HCC1143
**PI3K/mTOR**	**BEZ235**	26.2 ± 5.5	8.0 ± 1.7	32.72 ± 8.8	11.7 ± 6.5
	**GSK2126458**	8.6 ± 2.9	1.2 ± 0.1	10.8 ± 60	12.3 ± 3.6
**mTOR**	**AZD8055**	12.9 ± 5.7	5.7 ± 0.9	28.5 ± 5.7	18.0 ± 6.4
	**AZD2014**	19.6 ± 1.8	4.8 ± 0.6	21.1 ± 2.5	18.9 ± 2.0
	**KU-0063794**	9.2 ± 2.1	2.5 ± 1.1	13.9 ± 2.8	14.9 ± 3.0
**PI3K**	**GDC-0941**	2.4 ± 1.3	-2.1 ± 1.2	9.5 ± 3.6	11.5 ± 1.3

### Comparison of viability assay using Alamar Blue and proliferation assay using thymidine incorporation

**Fig 7:**
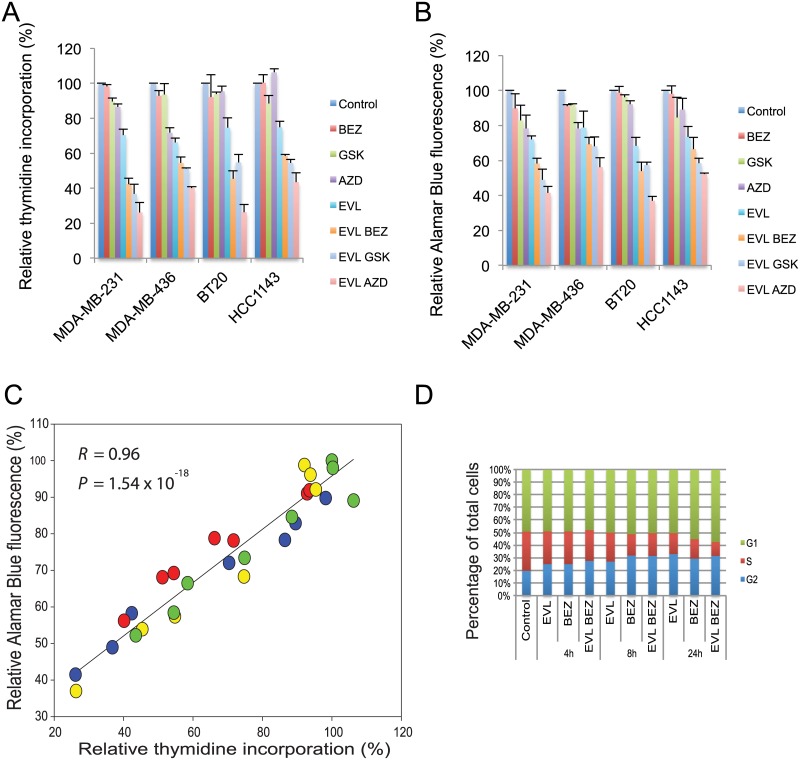
Comparison of viability assay using Alamar Blue and proliferation assay using thymidine incorporation, and the cell cycle changes in drug treatment. The inhibitory effects of drug combinations on MDA-MB-231, MDA-MB-436, BT20 and HCC1143 breast cancer cell lines were compared using (A) H^3^-thymidine incorporation assay and (B) Alamar Blue viability assay. BEZ, BEZ235 (10nM); GSK, GSK2126458 (2.5nM); AZD, AZD8055 (10nM); EVL, everolimus (100nM). Averages of three independent experiments are shown. (C) Scatter plot showing the relative thymidine incorporation (x-axis) and Alamar Blue fluorescence (y-axis). Significant correlation (Pearson Correlation, *R* = 0.96; Linear Regression, *P* = 1.54 x 10^−18^) was found between the thymidine uptake assay and viability assay in MDA-MB-231 (blue), MDA-MB-436 (red), BT20 (yellow); HCC1143 (green). Averages of two independent experiments are shown. (D) Cell cycle analysis of treated MDA-MB-231 cells. Cells were treated with DMSO control, 100 nM everolimus (EVL), 100nM BEZ235 (BEZ), everolimus and BEZ235 (EVL BEZ) combination. Cell number in each phase of the cell cycle was determined and calculated as a percentage of the total cell population.

We next assessed drug effects using Alamar Blue (to assess cell viability) and compared them with the results of the thymidine incorporation assay (which measures DNA synthesis) (Fig 7A and 7B). Viability assays in the four cell lines treated by single drug alone or combination using everolimus with BEZ235, GSK2126458 and AZD8055, respectively showed an excellent correlation (r = 0.96; *P* = 1.54 x 10^−18^) (Fig 7C).

### Mechanism of growth inhibitory action of everolimus and BEZ235

In a third method to investigate the effects of the inhibitors, MDA-MB-231 cells were treated with everolimus, BEZ235, or the combination and analyzed by flow cytometry. A time dependent decrease in the proportion of S-phase cells was observed in the treatment groups as compared to the control using (Fig 7D), consistent with the previous results (Fig 7A and 7B).

### Tests for synergy using combinations of everolimus with specific mTOR inhibitors (KU-0063794 and AZD2014) or the pan-PI3K inhibitor (GDC-0941)

**Fig 8:**
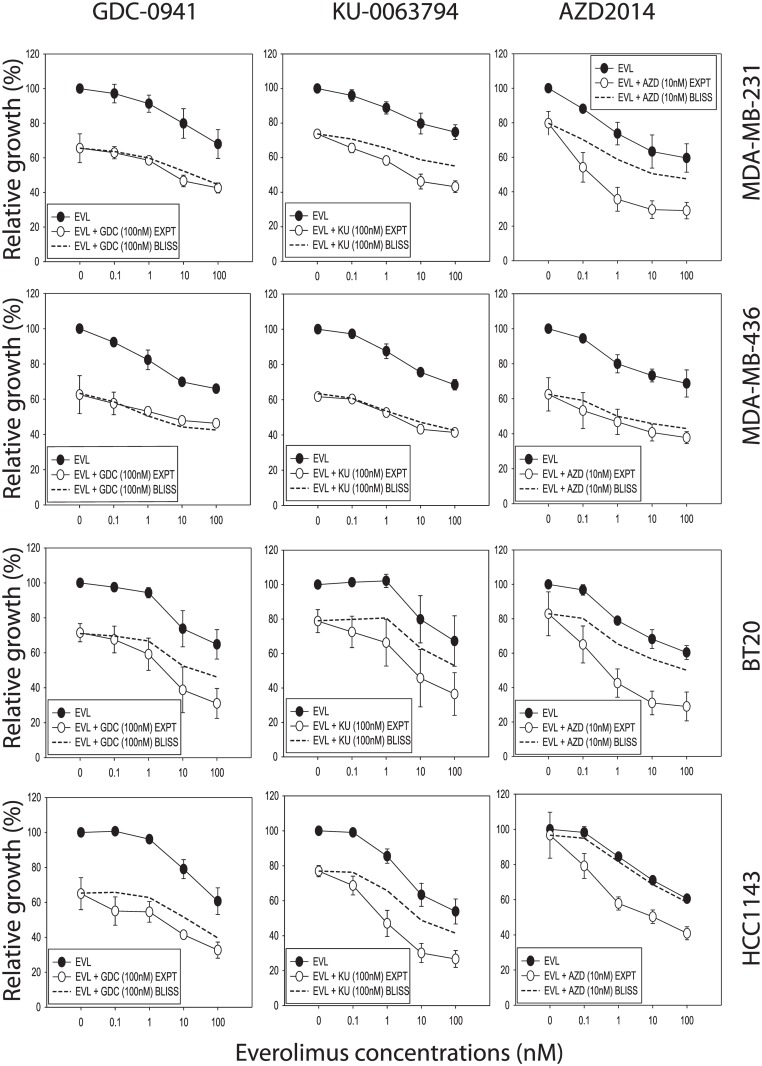
The growth inhibitory effects of drug combinations on MDA-MB-231, MDA-MB-436, BT20 and HCC1143 breast cancer cell lines. Growth inhibitory effects of combinations of everolimus with GDC-0941 (GDC) (left hand panel), KU-0063794 (KU) (middle panel) and AZD2014 (AZD) (right hand panel) using the Bliss additivism method. Dashed line, Bliss additivity curve, representing the theoretical expectation if the combined effects of everolimus with kinase inhibitors were exactly additive. Averages of three independent experiments are shown.

The synergism in growth inhibition observed with the above drug combinations indicated that targeting mTOR alone is sufficient for sensitizing the cell lines to everolimus. We therefore applied the same strategy to test the effects of two other mTOR specific inhibitors (KU-0063794 [6], AZD2014 [10]) and one pan-PI3K specific inhibitor (GDC-0941 [17]). Synergy was observed in all combinations tested, regardless of whether compounds were mTOR specific or PI3K specific (Fig 8). This result suggested that everolimus, at concentrations that did not inhibit proliferation by itself, increased the growth inhibitory effects of other drugs acting on the same signaling pathways.

**Fig 9:**
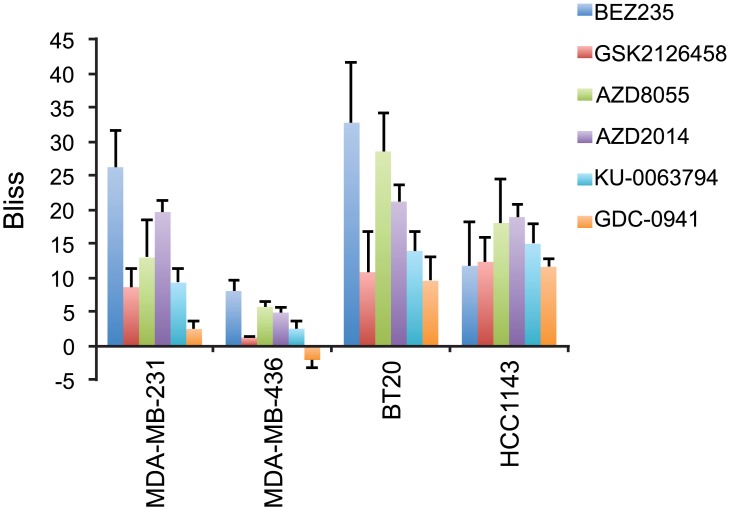
Growth sensitivity to the combination of everolimus and kinase inhibitors. Synergy, positive Bliss value, was observed for all drugs tested. Bliss = 0 indicates the combination is additive; Bliss > 0 indicates the percentage increase in maximal inhibition above additivity (synergy); and Bliss < 0 indicates the percentage increase in maximal inhibition below additivity (antagonism).

Although synergy was observed in all cell lines tested, the level of synergy of drug combination (everolimus and BEZ235, GSK2126458, AZD8055, AZD2014, KU-0063794 or GDC-0941) (Table 1; Fig 9) was found to be cell line specific. Significant differences in drug combination effects (Holm-Sidak method) were observed in BT20 (*p* < 0.01), HCC1143 (*p* = 0.01) and MDA-MB-231 (*p* = 0.023) using multiple comparisons and MDA-MB-436 as the control group.

## Discussion

In a broad series of breast cancer cell lines, sensitivity to the mTOR allosteric inhibitor everolimus, as measured by IC_50_ values, varied over a range of more than 570-fold. Sensitivity did not seem to be related to *PIK3CA* mutational status or ER status (Fig 2), in agreement with a previous study reporting a lack of correlation of everolimus sensitivity to *PIK3CA* mutation in breast cancer [28]. Triple-negative status partially stratified cell lines for everolimus resistance, although two triple-negative cell lines showed sensitivity, with IC_50_ values below 8 nM. Sensitivity was significantly correlated with the phosphorylation level of p70S6K (mTOR effector) (*p* = 0.005), but not with the phosphorylation of AKT or ERK, consistent with the mTOR pathway being a principal target of action of this drug.

The ER+ MCF-7 line showed high sensitivity to the mTOR inhibitor everolimus (Fig 2), possibly because the gene encoding p70S6K is amplified with corresponding overexpression and phosphorylation of the p70S6K protein [29]. The derived triple-negative MCF-7 endocrine therapy-resistant sub-lines FulvR1a, FulvR2a, and FulvR1c were also sensitive, with IC_50_ values 2.2, 2.8, and 3.1 nM, respectively, suggesting high ER expression is not required for everolimus response. Other mechanisms may explain the clinical observation that inhibition of ER can sensitize breast cancer cells to everolimus [30].

The main aim of our study was to examine growth inhibitory effects of combinations of inhibitors. We found that inhibitors targeting the same PI3K/mTOR pathway sensitize everolimus resistant breast cancer cell lines to growth inhibition (Fig 5), regardless of whether they are dual PI3K/mTOR kinase inhibitors (BEZ235 and GSK2126458) or an mTOR kinase specific inhibitor (AZD8055). Our result agrees with our previous report that everolimus and BEZ235 synergistically decrease proliferation in the triple-negative MDA-MB-231 cell line and in the ER+ MCF-7 sub-lines (TamC3 and TamR3) [31]. Strong synergy between everolimus and BEZ235 was also reported in various cancer cell lines with different lineages and genetic backgrounds [32].

The signaling response of concurrent targeting of both PI3K and mTOR pathways, as measured by phosphorylation of AKT and p70S6K respectively, did not predict growth inhibitory effects in MDA-MB-231, MDA-MB-436, BT20 and HCC1143 breast cancer cell lines. Everolimus alone efficiently inhibited the p70S6K pathway downstream of mTORC1, and activated AKT phosphorylation only in the HCC1143 cell line. Due to a negative feedback loop [33], inhibition of mTORC1 can induce AKT S473 phosphorylation in a subset of cancer cell lines and patient tumors [34–36], and therefore activation of AKT S473 might attenuate tumor responses [33,37]. Targeting mTORC1 alone with everolimus leads to consistent feedback activation of AKT while the dual mTORC1-2/PI3K inhibitor BEZ235 eliminates this feedback loop in breast cancer cells, yet both molecules are equally effective in inhibiting cell proliferation *in vitro* and *in vivo* despite these important signaling differences [38]. Here, as compared to BEZ235, both GSK2126458 and AZD8055 efficiently suppressed phosphorylation of AKT either alone or in combination with everolimus at the concentration tested (Fig 6). The attenuation of AKT S473 showed no correlation with the synergistic effects of growth inhibition as measured by Bliss values (Figs 6 and 9, Table 1). Our results agree with a report that the modulation of AKT phosphorylation by PI3K/mTOR inhibitors does not predict cell viability [15].

Since the mTOR pathway regulates protein translation [39] and mTOR inhibitors can impair synthesis of proteins encoded by mRNAs that contain a 5’-TOP (5’-terminal oligopyrimidine tract) [40], the synergistic effect in growth inhibition could also be due to the inhibition of synthesis of multiple proteins that are each critical for cell proliferation. We have investigated the growth inhibitory action of everolimus and BEZ235 in the MDA-MB-231 cell line, by flow cytometry. Everolimus (100 nM) decreased the proportion of S-phase cells in a time-dependent fashion over 24 hours and the effect was larger in combination with BEZ235 (100 nM), consistent with the concept that both of these drugs act by inhibiting the synthesis of proteins required for entry of cells into the S-phase of the cell cycle.

In this study, MDA-MB-436 showed the least utilization of the mTOR signaling pathway (as determined by phosphorylation of the signaling components p70S6K, rp-S6, and 4E-BP1), and also showed least synergism (in terms of growth inhibition) when treated with drug combinations (Figs 4, 5 and 9). Cellular dependence on mTOR pathway activity may be required for synergism to occur, and cells that are reliant on mTOR signaling for survival may respond more strongly to concurrent targeting of the mTOR pathway.

We also examined the growth inhibitory effect of a pan-PI3K inhibitor (GDC-0941), in comparison with inhibitors of mTOR specific kinase (AZD2014 and KU-0063794) to determine whether the observed synergistic effects are PI3K independent. Synergy was previously observed with the mTOR selective inhibitor KU-006394 [6,41]. GDC-0941 showed relatively lower synergistic activity in drug combination as compared with mTOR kinase inhibitors AZD2014 and KU-0063794, with weak antagonistic effects in MDA-MB-436 cells in some concentrations tested (Figs 7 and 8). Our results can be compared to a recent study in hepatocellular carcinoma, which showed that combination of everolimus with the PI3K specific inhibitor BKM120 exhibited weak antagonism, while combination of everolimus with BEZ235 showed weak synergism [42]. They can also be compared to the results of our previous study using the combinations of MEK inhibitor (trametinib) and PI3K/mTOR inhibitors (everolimus, BEZ235 or GSK2126458), where cell line-specific synergism was also observed. It was proposed that BEZ235 could suppress a negative feedback loop mediated by mTORC2, thereby leading to enhanced MEK/ERK pathway activity [43]. We observed increased ERK phosphorylation in BT20 cell line, although it has no correlation with the synergism observed in the combination of everolimus and BEZ235.

It was suggested that reducing the degree of mTORC1 signaling (as assessed by the phosphorylation of substrates) would decrease the drug concentration required to fully inhibit mTORC1 activity [32]. While inhibition of signaling pathways with small molecule inhibitors as single agents can be efficacious, anti-tumor activity might be better achieved by concurrent treatment with multiple drugs affecting the same signaling pathway. For example, the combination of allosteric and ATP-competitive inhibitors of Bcr-Abl can maximize target inhibition, and combining allosteric and ATP-competitive inhibitors can suppress resistance to either agent alone [44]. Everolimus is relatively well tolerated in patients, but it is currently restricted to treat ER+ breast cancer in the clinic. We have demonstrated that the combination of sub-optimal concentrations of PI3K/mTOR, pan-PI3K or mTOR ATP-competitive inhibitors and everolimus can achieve synergistic inhibition of the proliferation of human breast cancer cells *in vitro*. Our results provide the rationale to design *in vivo* studies in the future, which may represent a novel strategy to enhance the efficacy of mTOR-targeted breast cancer therapy. Measurement of p70S6K phosphorylation can potentially identify human breast cancers that would benefit from therapy with the mTOR allosteric inhibitor everolimus. The suitability of such candidate biomarkers will need to be confirmed in future clinical trials.

## Supporting Information

S1 Fig Signaling pathway usage as measured by basal protein phosphorylation of ERK in MDA-MB-231, MDA-MB-436, BT20 and HCC1143 cell lines. Immunoblots with antibodies specific for phosphorylated ERK proteins and total ERK protein are indicated. Actin is the loading control.
(EPS)

S2 Fig The cellular response to drug combinations of MDA-MB-231 and MDA-MB-436 breast cancer cells. Cells were treated with drugs for 24 h and signaling pathway usage was measured by protein phosphorylation of AKT, p70S6K, rpS6, 4E-BP1 and ERK in the four cell lines. Immunoblots with antibodies specific for phosphorylated proteins and total proteins are indicated. Actin is the loading control.
(EPS)

S3 Fig The cellular response to drug combinations used to treat BT20 and HCC1143 breast cancer cells. Cells were treated with drugs for 24 h and signaling pathway usage were measured by protein phosphorylation of AKT, p70S6K, rpS6, 4E-BP1 and ERK in the four cell lines. Immunoblots with antibodies specific for phosphorylated proteins and total proteins are indicated. Actin is the loading control.
(EPS)
